# Two Cases of Small Intestinal Follicular Lymphoma Presenting with Intestinal Stricture

**DOI:** 10.70352/scrj.cr.25-0108

**Published:** 2025-06-18

**Authors:** Akihiro Nakamura, Syuichi Komori, So Murai, Shiori Shibata, Hideyuki Oyama, Kazuhiro Kijima, Yoshikuni Harada, Gaku Kigawa, Takahiro Umemoto, Takafumi Ogawa, Kuniya Tanaka

**Affiliations:** 1 Department of General and Gastroenterological Surgery, Showa Medical University Fujigaoka Hospital, Yokohama, Kanagawa, Japan; 2 Department of Pathology and Laboratory Medicine, Showa Medical University Fujigaoka Hospital, Yokohama, Kanagawa, Japan

**Keywords:** follicular lymphoma, small intestine, stricture, obstruction, lymphoma

## Abstract

**INTRODUCTION:**

Primary gastrointestinal follicular lymphoma (FL) rarely causes intestinal stricture. We report two cases of small intestinal FL presenting with stricture.

**CASE PRESENTATION:**

Case 1: A 63-year-old man presented with small intestinal obstruction. CT demonstrated ileal wall thickening and enlarged lymph nodes. Partial ileal resection confirmed primary ileal FL, immunohistochemically positive for CD10, CD20, and BCL-2. Case 2: A 79-year-old woman with a 7-year history of jejunal strictures underwent right hemicolectomy for ascending colon cancer and partial jejunal resection. Pathologic examination showed concurrent jejunal FL and colon adenocarcinoma. Immunohistochemical findings were the same as in Case 1. In both patients, postoperative positron-emission tomography-CT showed no residual lymphoma. Both were monitored clinically without chemotherapy.

**CONCLUSIONS:**

These cases highlight an unusual presentation of follicular lymphoma as a cause of intestinal stricture. Surgical resection provided diagnostic clarity and relief of symptoms. Postoperative treatment was tailored to individual patient characteristics and residual disease status.

## Abbreviations

DLBCL
: diffuse large B-cell lymphoma
FISH
: fluorescence *in situ* hybridization
FL
: follicular lymphoma
HGBCL
: high-grade B-cell lymphoma
NHL
: non-Hodgkin lymphoma
PI-NHL
: primary intestinal non-Hodgkin lymphoma

## INTRODUCTION

FL is the second most common subtype of NHL, but primary gastrointestinal involvement is relatively uncommon. Such FL most commonly occurs in the duodenum, but can arise more distally in the small intestine.^1,2)^ FL typically presents as multiple small nodular lesions in the submucosa and usually follows an indolent clinical course.^3,4)^ Intestinal stricture is a rare presentation of FL, as the tumor is soft and characteristically grows submucosally without tissue invasion or destruction.^5)^ Unlike DLBCL, which commonly presents as mass-forming lesions that can cause mechanical obstruction, FL rarely causes luminal stricture or obstruction.^1)^ The few reported cases of FL-related stricture were usually associated with either long-standing disease progression or extensive circumferential involvement.^5)^

Here, we report two patients with small intestinal FL presenting as intestinal stricture, highlighting the importance of FL as a rare but important cause of small bowel obstruction.

## CASE PRESENTATION

### Case 1

A 63-year-old man presented with nausea and vomiting. He had no past history of abdominal surgery. Family history was unremarkable. Physical examination disclosed abdominal distension without tenderness. Vital signs were temperature: 36.4°C; blood pressure: 125/82 mmHg; pulse rate: 73 beats per minute; and oxygen saturation: 99% on room air.

Laboratory results included white blood cell count (WBC): 6750/μL; hemoglobin (Hb): 16.4 g/dL; platelet count (PLT): 30.5 × 10^4^/μL; albumin (Alb): 4.2 g/dL; creatinine (Cre): 0.9 mg/dL; sodium (Na): 142 mEq/L; chlorine (Cl): 102 mEq/L; potassium (K): 4.2 mEq/L; aspartate aminotransferase (AST): 21 U/L; alanine transaminase (ALT): 14 U/L; and C-reactive protein (CRP): 1.83 mg/dL. Tumor marker concentrations were carbohydrate antigen (CA) 19-9: 12.1 U/mL (normal range: ≤37.0 U/mL); carcinoembryonic antigen (CEA): 4.1 ng/mL (normal range: ≤5.0 U/mL); and serum soluble interleukin-2 receptor: 312 U/mL (normal range: 122–496 U/mL).

**Fig. 1:**
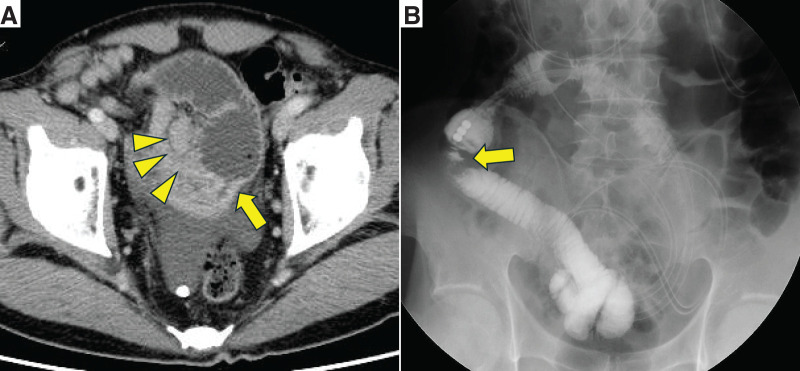
(**A**) Contrast-enhanced CT of the abdomen in case 1 showed thickening of the ileal wall with contrast effect, dilation of the proximal bowel (arrow), and enlarged lymph nodes near the ileal tract (arrowheads). (**B**) A small intestinal series performed using the ileus tube showed fully circumferential stricture extending for 18 mm (arrow).

Abdominal contrast-enhanced CT showed thickening of the ileal wall with contrast effect, proximal bowel dilation, and enlarged lymph nodes near the ileal tract (**Fig. 1A**). Small intestinal obstruction was managed by decompression using an ileus tube. A subsequent small intestinal series with contrast administered via the ileus tube showed a pan-circumferential stricture 18 mm in length (**Fig. 1B**).

After bowel movements resumed and abdominal distension improved, the ileus tube was removed and meals were resumed, but intestinal obstruction recurred. Since conservative treatment was unsuccessful, laparoscopic partial resection of the small intestine was performed 44 days after admission, followed by end-to-end anastomosis. Intraoperatively, a firm mass was detected in the ileum, and mesenteric lymph node enlargement was noted. Partial resection of the small intestine was performed, including as many enlarged mesenteric lymph nodes as possible.

**Fig. 2:**
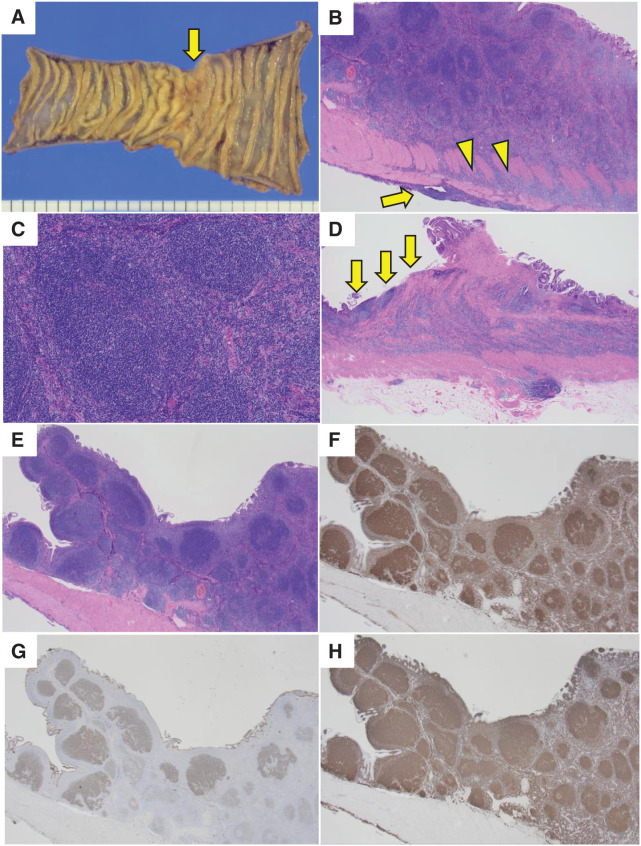
(**A**) The specimen from the resected ileum showed circumferential wall thickening with ulcerative lesions (arrow). (**B**) HE staining viewed with a 2× objective lens showed nodular structures. Atypical lymphocytes infiltrated the serosal layers (arrow) and invaded the neural plexus (arrowheads). (**C**) HE staining (10× objective lens) showed proliferation of small- to medium-sized atypical lymphocytes. (**D**) HE staining (2× objective lens) showed an ulcerative lesion (arrows). (**E**) HE staining of nodules (2× objective lens). In panels (**F**–**H**), immunohistochemical analysis showed centers of nodules to be positive for Bcl-2, CD10, and CD20 respectively; 2× objective lens. HE, hematoxylin-eosin

Pathologic examination of the resected specimen (**Fig. 2**) showed circumferential wall thickening with ulcerative lesions and nodular proliferation of small- to medium-sized atypical lymphocytes, primarily in the submucosal layer. Fibrosis of intestinal wall stroma was not prominent. Similar dense proliferations of atypical lymphocytes in a nodular pattern were evident within mesenteric lymph nodes. Centroblasts numbered 1 to 5 per high-power field (hpf; objective lens: 40×), and Ki-67 immunostaining was present in about 15% of cells. Diffuse infiltrates of atypical lymphocytes surrounded these nodules. Atypical lymphocytes infiltrated the muscularis and serosa with invasion extending into the neural plexus. Immunohistochemical analysis showed that cells in the centers of the nodules were positive for CD10, CD20, CD21, BCL-2, and BCL-6, but negative for CD3. FISH showed a positive BCL2 split signal.

Based on these findings, the patient was diagnosed with primary ileal FL. Postoperative PET-CT showed no evidence of residual lymphoma lesions. The clinical stage of disease according to the Lugano staging system for gastrointestinal lymphomas^6)^ was II_1_, and by the WHO classification^7)^ the disease was Classic FL. The patient was discharged on postoperative day 9 without complications.

He is currently being monitored as an outpatient, with no antineoplastic drug treatment.

### Case 2

**Fig. 3:**
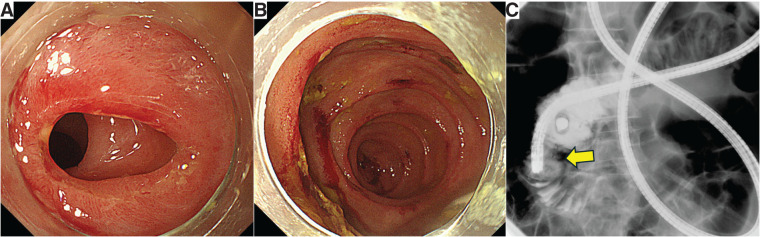
(**A**) Enteroscopy showed strictures in the jejunum. (**B**) Enteroscopy showed multiple erosions in the jejunum. (**C**) Small intestinal series performed using the ileus tube showed a stricture involving the entire circumference for a length of 16 mm (arrow).

A 79-year-old woman presented 7 years prior to this report with recurrent vomiting, diarrhea, and abdominal pain. Enteroscopy disclosed strictures (**Fig. 3A**) and multiple ulcerations in the jejunum (**Fig. 3B**). A small intestinal series performed with contrast medium given via ileus tube showed stricture involving the entire circumference over a length of 16 mm (**Fig. 3C**). Balloon dilation was performed. Microscopic specimens from ulceration and stricture sites showed nonspecific inflammatory changes. A small intestinal type of Crohn’s disease was initially suspected; she was treated with mesalazine, budesonide, and ustekinumab, as her course was monitored. The ulcerative lesion improved, while strictures remained stable without progression for 6 years until she returned because of worsening diarrhea, abdominal pain, and anorexia persisting over a month. The patient’s past history included appendectomy, cholecystectomy, and surgery to treat ileus. Family history was unremarkable. Physical examination was notable for mild abdominal distension without tenderness. Vital signs were temperature: 36.4°C; blood pressure: 99/66 mmHg; and pulse rate: 83 beats per minute. Oxygen saturation was 97% on room air.

Laboratory test results included WBC: 9270/μL; Hb: 10.2 g/dL; PLT: 31.0 × 10^4^/μL; Alb: 3.7 g/dL; Cre: 1.12 mg/dL; Na: 135 mEq/L; Cl: 103 mEq/L; K: 4.8 mEq/L; AST: 24 U/L; ALT: 11 U/L; and CRP: 2.59 mg/dL. Values for tumor markers were CA19-9: 25.2 U/mL; and CEA: 1.6 ng/mL.

**Fig. 4:**
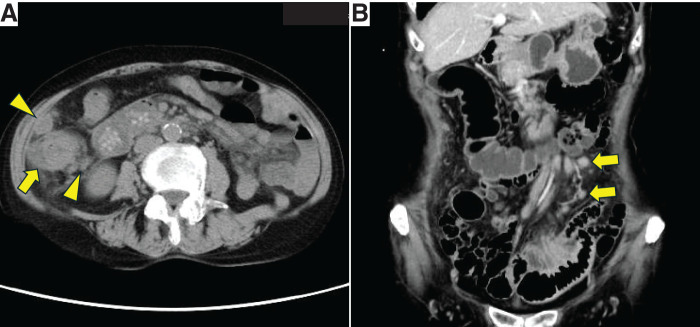
(**A**) Noncontrast CT of the abdomen showed thickening of the wall of the ascending colon (arrow) and enlargement of surrounding lymph nodes (arrowheads). (**B**) Contrast CT of the abdomen showed enlarged jejunal mesenteric lymph nodes (arrows).

Noncontrast CT of the abdomen (**Fig. 4A**) showed thickening of the wall of the ascending colon, enlargement of surrounding lymph nodes, and dilation of the proximal bowel. Colonoscopy showed a circumferential ulcerative lesion in the ascending colon associated with severe stricture. An ascending colon cancer with stricture was suspected, and colonic stenting was performed. Contrast-enhanced CT of the abdomen after stenting (**Fig. 4B**) showed enlargement of the jejunal mesenteric lymph nodes and para-aortic lymph nodes that had been noted 7 years previously, now showing no change in size. The nodes were considered to represent inflammatory lymphadenopathy caused by Crohn’s disease.

A biopsy specimen from the colon showed only necrosis, with no definitive histologic diagnosis. Nonetheless, resection was planned to treat suspected obstructive cancer of the ascending colon. Additionally, partial resection of the previously noted jejunal stricture was scheduled to establish its etiology as well as treat the obstruction.

Intraoperative observation determined that the ascending colon tumor had partially invaded the duodenum, and suspected tumor nodules were discovered in the omentum. Laparoscopic right hemicolectomy with partial duodenal resection and excision of omental nodules was performed. The jejunum showed stricture with proximal bowel dilation, and mesenteric lymph nodes were enlarged. Partial small bowel resection was performed, including as many mesenteric lymph nodes as possible.

**Fig. 5:**
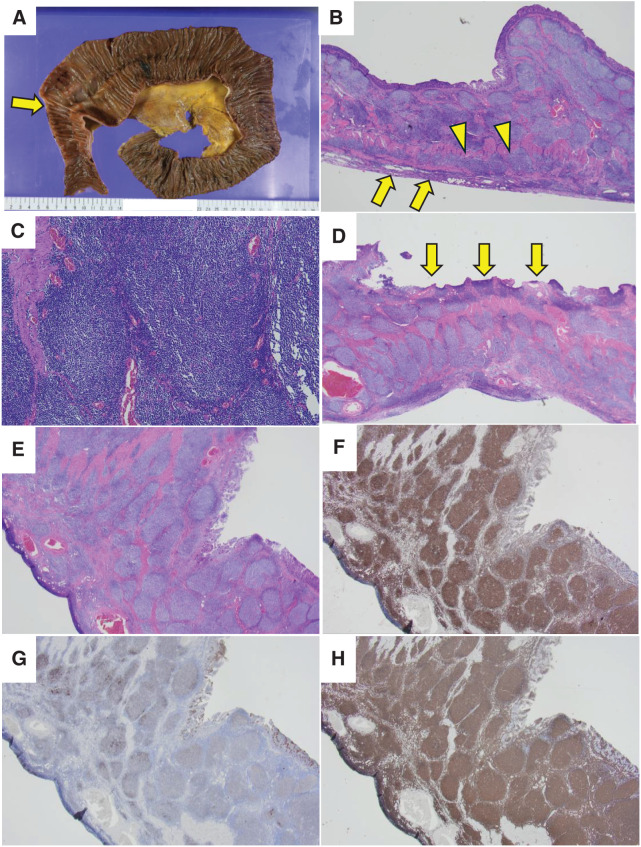
(**A**) The resected jejunal specimen showed circumferential wall thickening with ulcerative lesions and dilation of the proximal intestine (arrow). (**B**) HE staining, 2× objective lens: Nodular areas were observed. Atypical lymphocytes infiltrated serosal layers (arrows) and invaded the neural plexus (arrowheads). (**C**) HE staining, 10× objective lens: Proliferation of small-sized atypical lymphocytes was observed. (**D**) HE staining, 2× objective lens: Ulceration was observed (arrows). (**E**) HE staining, 2× objective lens showed centers of nodules. (**F**–**H**) Immunohistochemical analyses of the same field were positive for Bcl-2, CD10, and CD20 (**F**: Bcl-2 staining, 2× objective lens, **G**: CD10 staining, 2× objective lens, **H**: CD10 staining, 2× objective lens). HE, hematoxylin-eosin

Adenocarcinoma was diagnosed in the resected ascending colon specimen. Metastases were confirmed in the regional lymph nodes and omentum. The ascending colon cancer was assessed as Stage IV (T4aN2aM1c). The resected jejunal specimen (**Fig. 5**) showed circumferential wall thickening with ulcerative lesions and proliferation of small atypical lymphocytes in a nodular pattern throughout its thickness. Fibrosis of the intestinal wall stroma was not prominent, but atypical lymphocytes infiltrated the neural plexus. Similar dense proliferation of atypical lymphocytes in a nodular pattern was seen in mesenteric lymph nodes. Centroblasts numbered 1 to 5 per hpf (objective lens: 40×). Ki-67 was positive in 1%–5% of these cells. By immunohistochemical analysis centers of nodules were positive for CD10, CD20, CD21, BCL-2, and BCL-6, but negative for CD3. FISH showed a positive BCL2 split signal. Additionally, mesenteric lymph nodes adjoining the ascending colon showed areas of follicular proliferation with similar staining results, suggesting metastasis of follicular lymphoma.

Based on these findings, the jejunal lesion was diagnosed as primary ileal FL.

Postoperative PET-CT showed no evidence of residual lymphoma lesions. The lesion’s clinical stage according to the Lugano staging system for gastrointestinal lymphomas^6)^ was II_2_, and its WHO classification^7)^ was Classic FL. Although mild enlargement of the para-aortic lymph nodes was observed on preoperative imaging, these nodes were not surgically removed. Therefore, FL involvement cannot be definitively ruled out based on imaging alone. However, the size of the para-aortic lymph nodes had remained unchanged for 7 years before surgery, and there were no other signs of systemic disease. Based on this long-term stability, we consider FL involvement of the para-aortic lymph nodes to be unlikely. The patient was discharged on postoperative day 15 without complications. Chemotherapy is being administered to treat the colorectal cancer, while the follicular lymphoma is being monitored without further specific treatment.

## DISCUSSION

Among all NHL cases, 30%–40% are PI-NHL. Their most common site of occurrence is the stomach, followed by the small intestine. They account for 2%–24% of malignant small intestinal tumors.^8–10)^ Among PI-NHL histologic types, DLBCL is most common (45%), followed by FL (18%) and mucosa-associated lymphoid tissue (MALT) lymphoma (14%).^10)^ Regarding the histologic diagnosis of FL, the presence of a nodular architecture strongly suggests FL. Immunohistochemically, FL typically shows positivity for CD10, CD20, CD21, BCL2, and BCL6, and negativity for CD3.^11)^ By contrast, MALT lymphoma generally lacks follicular architecture and is negative for CD10 and BCL6, often exhibiting lymphoepithelial lesions and plasmacytic differentiation.^11)^ DLBCL is usually characterized by a diffuse architecture, marked cytologic atypia, and a high Ki-67 proliferation index; however, rare cases of high-grade transformation from FL require careful histologic grading and Ki-67 evaluation.^11)^

Intestinal obstruction is reported to occur in 5%–39% of gastrointestinal malignant lymphomas; intussusception occurs even more frequently, reportedly in 46% of such cases, particularly ileocecal lymphomas.^8,12)^ Histologic types of malignant lymphoma known to cause intestinal obstruction include DLBCL (43%), Burkitt lymphoma (22%), and mantle lymphoma (13%). Intestinal obstruction occurs in 46% of DLBCL and 15% of Burkitt lymphoma cases.^12,13)^ On the other hand, although FL is the second most common histologic type of PI-NHL, it accounts for only 5% of malignant lymphomas causing intestinal obstruction.^13)^ FL rarely causes intestinal obstruction because of its usual mucosal and submucosal location, low invasiveness, slow progression, paucity of fibrosis, and soft consistency.^3,5,13–15)^ However, when increasing numbers of tumor cells infiltrate the small intestinal wall, FL can cause wall thickening and ulceration, likely to lead to stricture.^16)^ Firmness of the tumor is also influenced by cell density^17)^; increased cell density can raise pressure within the tumor, increasing firmness despite the absence of fibrosis. In the present case, dense proliferation of small lymphocytes may have hardened the tumor.

In our cases, we found circumferential wall thickening, tumor ulceration, and extension into the muscularis and nerve plexus, but no notable stromal fibrosis. We suspect that intestinal obstruction resulted from both mechanical luminal stenosis and functional peristaltic disturbance caused by muscle and nerve damage. While infiltration beyond the muscular layer is considered rare, specific frequency data are unavailable. Paucity of data can be attributed to the rarity of primary gastrointestinal FL and the fact that many asymptomatic patients undergo biopsy without resection, impeding assessment of invasion depth.

The Lugano classification^6)^ is the usual basis for staging gastrointestinal lymphomas. Stages I and II_1_ are limited to regional lymph node metastasis, representing limited-stage disease, while Stages II_2_ and above denote advanced disease. No clear treatment guidelines for primary gastrointestinal FL have been established. Treatment is generally based on whether disease is limited or advanced. While drug therapy alone sometimes is used for localized lesions, Watanabe^18)^ states that surgical resection should be the first choice for localized lesions, followed by drug therapy. However, some reports show no difference in outcome between treatment and observation for Stage I disease, so in practice, “watchful waiting” is often used at this limited stage.^19)^ While several reports advocate radio therapy for duodenal lesions, irradiation of the small intestine is uncommon due to concern about adverse events such as stenosis.^20)^ At the minimum, surgical resection should be considered for limited-stage lesions that are symptomatic, causing pain, stenosis, intussusception, or bleeding.^21)^ Another advantage of resection is that it permits a pathologic diagnosis even when the preoperative diagnosis is unclear. Both of our cases were diagnosed by examination of resected specimens, while no preoperative diagnosis could be made. While some reports show higher complete remission rates and overall survival rates when post-resection chemotherapy is given for NHL, other reports dealing specifically with FL noted that more than half of the patients studied underwent only postoperative observation without chemotherapy. No consensus therefore exists as to whether chemotherapy should be added after resection of primary gastrointestinal FL.^10,22,23)^ For non-resected advanced-stage cases, combination chemotherapies including rituximab such as R-CHOP (Rituximab, Cyclophosphamide, Doxorubicin, Vincristine, and Prednisone) or R-CVP (Rituximab, Cyclophosphamide, Vincristine, and Prednisone) are commonly used.^17,20)^

Overall, the prognosis for GI-FL is good; Matysiak-Budnik et al.^20)^ have reported that over 80% of patients were still alive 10 years after diagnosis, with 70% of them showing no disease progression. Chouhan et al.^23)^ reported that small intestinal cases in particular had a better prognosis with 5-year overall survival (OS), at 80.9% compared with FL occurring in the stomach (5-year OS, 52.7%) or colon (5-year OS, 71.5%). However, approximately 7% of cases progress to become higher-grade lymphomas such as DLBCL or HGBCL, a possibility necessitating careful follow-up.^24)^ Although no standardized protocol exists specifically for GI-FL, recommendations for nodal FL by the European Society for Medical Oncology suggest structured surveillance following the end of therapy.^25)^ During the first 2 years, follow-up should include history-taking, physical examination, and blood tests every 3–6 months. Imaging studies, including abdominal ultrasound or CT should be performed every 6–12 months if clinically indicated. In years 3–5, the frequency may be reduced to every 6–12 months, and annually thereafter.

To date, 14 cases of GI-FL presenting with small bowel stenosis have been reported in English, including our present cases (**Table 1**).^16,26–35)^ These cases show a slight male predominance. Most patients presented with symptoms such as abdominal pain and vomiting. The jejunum was the most common site. Among 11 patients who underwent resection, only 3 were diagnosed with FL preoperatively. As small bowel stricture caused by FL is rare, this neoplasm is unlikely to be considered in a differential diagnosis. Endoscopic examination generally is difficult to perform in patients with bowel obstruction. Furthermore, the small intestine is largely beyond the reach of conventional gastrointestinal endoscopic screening. For these reasons, preoperative diagnosis can be impossible. According to macroscopic appearance, Nakamura et al. classified small intestinal malignant lymphomas as superficial, polypoid, ulcerative, polyposis, diffuse, or mixed types; the ulcerative type is rare, accounting for only 2.5% of small intestinal FL cases.^36–38)^ In all eight cases, including our own where macroscopic or endoscopic findings were described, circumferential ulceration was present. However, to date, no previous studies have systematically reviewed the macroscopic appearance of small intestinal FL. We believe that our report provides valuable insight into the gross morphological features of FL presenting with bowel stricture, an aspect that has been largely overlooked in the existing literature. As few previous reports have addressed the mechanisms of stricture formation in detail, it remains unclear whether ulceration is directly responsible. Further accumulation of cases and detailed pathological analysis are needed to clarify this issue. Of the four cases where the extent of tumor invasion was described, including our own, diffuse infiltration beyond the muscular layer had occurred. High-grade transformation to DLBCL was noted in a case reported by Nishimura et al.,^29)^ with Ki-67 positivity exceeding 80%. Among cases undergoing resection, four were managed with postoperative observation without chemotherapy, while six received chemotherapy. Among reports where survival outcomes were stated, no disease-specific deaths were reported. These findings suggest that resection is often performed in cases with stricture for both therapeutic and diagnostic purposes. Either observation or chemotherapy may be chosen after surgery based on details of the case, leading to favorable prognoses. Increasing use of double-balloon endoscopy (DBE) and capsule endoscopy is expected to increase the diagnostic rate of small intestinal FL. In addition to improving the diagnostic yield, DBE also enables therapeutic interventions such as endoscopic balloon dilation (EBD) for FL-related strictures. Although EBD is generally contraindicated in malignant strictures due to the risk of perforation, selected cases have been successfully managed with DBE-assisted EBD under strict procedural criteria such as the absence of deep ulceration and short stricture length.^31)^ This approach may be a viable option for poor surgical patients. By contrast, self-expandable metallic stents, while widely used in colorectal cancer, have not been reported for small intestinal FL. Given the risk of perforation from long-term placement, stenting should be reserved for exceptional cases, such as for preoperative decompression or palliation. Accumulation of more cases should help clarify indications for resection, the role of postoperative chemotherapy, and the potential use of endoscopic interventions.

**Table 1:** Case reports of follicular lymphoma presenting with intestinal stricture

No.	Author	Year	Age (years)	Sex	Symptoms	Location	Resection	Diagnostic procedure	Ulceration	WHO grade	Diffuse infiltration	Ki-67 (%)	Lugano stage	Postoperative chemotherapy	Prognosis (months)	
1	Yamada^26)^	2016	72	F	Anemia	Ileum	Absent	Biopsy	Present	N/A	N/A	N/A	II_2_	N/A	36	Alive
2	Kawasaki^27)^	2016	63	F	Vomitting	Jejunum	Present	Resection	N/A	1	Present	N/A	II_2_	Present	N/A	
3	Pezzella^28)^	2018	64	M	Epigastric pain	Ileum	Present	Resection	N/A	1–2	N/A	N/A	N/A	Present	6	Alive
4	Nishimura^29)^	2018	82	F	Vomitting	Jejunum	Present	Biopsy	Present	N/A	Present	81	N/A	Present	N/A	
5	Kawasaki^30)^	2019	77	M	Abd pain and vomitting	Ileum	Present	Resection	Present	N/A	N/A	N/A	N/A	Present	N/A	
6	Magome^31)^	2020	60	M	N/A	Jejunum	Absent	N/A	N/A	N/A	N/A	N/A	II_1_	N/A	N/A	
7			68	M	N/A	Jejunum	Absent	N/A	N/A	N/A	N/A	N/A	II_1_	N/A	N/A	
8	Delgado^32)^	2021	35	M	Abd pain and vomitting	Jejunum	Present	Resection	N/A	N/A	N/A	N/A	N/A	N/A	N/A	
9	Osaki^33)^	2021	73	F	Abd pain and nausea	Ileum	Present	Resection	Present	N/A	N/A	N/A	N/A	Absent	4	Alive
10	Goto^34)^	2022	79	M	Abd pain and vomitting	Jejunum	Present	Biopsy	Present	1	Present	N/A	II_2_	Present	12	Alive
11	Suda^35)^	2022	60	F	Epigastric pain and vomitting	N/A	Present	Resection	N/A	3A	N/A	N/A	N/A	Present	N/A	
12	Suzuki^16)^	2022	73	M	Vomitting	Jejunum	Present	Resection	Present	N/A	N/A	N/A	II_1_	Absent	24	Alive
13	Our case	2024	63	M	Vomiting	Ileum	Present	Resection	Present	1	Present	15	II_1_	Absent	5	Alive
14		2024	79	F	Diarrhea, abd pain and anorexia	Jejunum	Present	Resection	Present	1	Present	1–5	II_2_	Absent	3	Alive

abd, abdominal; F, female; M, male; N/A, data not available

## CONCLUSIONS

This case highlights an unusual presentation of small intestinal follicular lymphoma causing intestinal stricture. While gastrointestinal lymphomas rarely cause strictures, lymphoma should be considered in the differential diagnosis of small bowel stricture. Since endoscopic screening remains difficult to perform in the small intestine, resection of the stricture site when appropriate provides an opportunity for pathologic diagnosis that will help to guide treatment choices after resection.

## ACKNOWLEDGMENTS

We thank Dr. Yosuke Sasaki for providing us with pathological analysis including FISH, and Dr. Jeffrey Smith for editing assistance.

## DECLARATIONS

### Funding

No funding was received for this study.

### Authors’ contributions

A.N., S.K., and K.T. participated in report conceptualization and draft writing.

S.S., H.O., K.K., Y.H., G.K., and T.U. managed the patients.

A.N., S.M., and T.O. participated in pathologic analysis.

All authors have read and approved the manuscript and agree to be held accountable for all aspects of this report.

### Availability of data and materials

The datasets supporting the conclusions of this study are included in this article and its additional files.

### Ethics approval and consent to participate

This study was approved by our Institutional Review Board (CR2024046-A). Informed consent to participate in this study was obtained from the patients.

### Consent for publication

Consent was obtained from the patients for the publication of this case report.

### Competing interests

All authors declare no competing interests for this article.
